# Genetic Structure and *Wolbachia* Genotyping in Naturally Occurring Populations of *Aedes albopictus* across Contiguous Landscapes of Orissa, India

**DOI:** 10.1371/journal.pone.0094094

**Published:** 2014-04-08

**Authors:** Biswadeep Das, Truptimayee Satapathy, Santanu K. Kar, Rupenangshu K. Hazra

**Affiliations:** Department of Medical Entomology, Regional Medical Research Centre, Bhubaneswar, Odisha, India; University of Poitiers, France

## Abstract

**Background:**

*Aedes albopictus* has recently been implicated as a major vector in the emergence of dengue and chikungunya in several parts of India, like Orissa, which is gradually gaining endemicity for arboviral diseases. *Ae. albopictus* is further known to be naturally infected with *Wolbachia* (maternally inherited bacterium), which causes cytoplasmic incompatibility (CI) in mosquitoes leading to sperm-egg incompatibility inducing the death of embryo. Knowledge of genetic diversity of *Ae. albopictus*, along with revealing the type of *Wolbachia* infection in *Ae. albopictus* is important to explore the genetic and biological characteristics of *Ae. albopictus*, prior to exploring the uses of CI-based vector control strategies. In this study, we assessed the population genetic structure and the pattern of *Wolbachia* infection in *Ae. albopictus* mosquitoes of Orissa.

**Methods and Results:**

*Ae. albopictus* mosquitoes were collected from 15 districts representing the four physiographical regions of Orissa from 2010–2012, analyzed for genetic variability at seven microsatellite loci and genotyped for *Wolbachia* strain detection using *wsp* gene primers. Most microsatellite markers were successfully amplified and were polymorphic, showing moderate genetic structure among all geographic populations (F_ST_ = 0.088). Genetic diversity was high (F_ST_ = 0.168) in Coastal Plains populations when compared with other populations, which was also evident from cluster analyses that showed most Coastal Plains populations consisted of a separate genetic cluster. Genotyping analyses revealed that *Wolbachia*-infected *Ae. albopictus* field populations of Orissa were mostly superinfected with wAlbA and wAlbB strains. *Wolbachia* superinfection was more pronounced in the Coastal Plain populations.

**Conclusion:**

High genetic structure and *Wolbachia* superinfection, observed in the Coastal Plain populations of Orissa suggested it to be genetically and biologically more unique than other populations, and hence could influence their vectorial attributes. Such high genetic diversity observed among Coastal Plains populations could be attributed to multiple introductions of *Ae. albopictus* in this region.

## Introduction

The Asian tiger mosquito, *Aedes albopictus* (Skuse 1884) is a highly invasive species and has been implicated as one of the major vectors of arboviral diseases in many parts of India. *Ae. albopictus* has been reported to be naturally infected with dengue and chikungunya virus during several outbreaks that flourished throughout India in recent past [1], [2]. *Ae. albopictus* exhibits strong physiological and ecological flexibility, allowing it to thrive in wide climatic ranges and habitats. Previously known to be forest dweller, *Ae. albopictus* has now adapted itself to human environments and is preferentially found in rural and suburban areas [3], although it has also been recorded in densely populated urban areas of many parts of India [4], [5]. The mosquito is a prolific day biter, exhibiting multiple feeding habits and preferentially feeds on mammals, apart from amphibians, -reptiles and birds [6]. The feeding preferences of *Ae. albopictus* vary with the geographical locations of mosquito populations [7]. The diverse host spectrum of *Ae. albopictus* enhances not only its biological traits like fecundity and survival, but also its capacity to propagate zoonotic pathogens across animal species and to humans, thereby rendering it to be a potential vector of arboviral diseases [8].

Exploring the pattern and magnitude of genetic differentiation among vector populations is essential to evaluate the risk of pathogen transfer [9], [10]. Mosquito vectors are generally known to have genetic variability with time and geographical landscapes, which leads to evolution of species that are capable of transmitting diseases more efficiently. Hence study of genetic differentiation in mosquito populations from different topographical regions is essential to reveal population division/structuring of vectors.

The maternally inherited bacterium *Wolbachia* (Family: Anaplasmataceae), which induces crossing sterility known as cytoplasmic incompatibility (CI) in arthropods [11], occurs naturally in *Ae. albopictus* [12]. CI occurs when non infected females cannot produce offspring when they mate with *Wolbachia* infected males. Thus, in host populations that include both infected and uninfected individuals, CI provides a reproductive advantage to infected females since they can mate successfully with all male types. However, the mating between male and female mosquitoes infected with different and incompatible strains of *Wolbachia* will lead to considerable reduction in fecundity and egg hatching rate [13]. Studies have shown that *Ae. albopictus* is naturally infected by two types of *Wolbachia*, A and B [11], [12]. Naturally occurring populations of *Ae. albopictus* can be single-infected with wAlbA/wAlbB strain or superinfected with wAlbA and wAlbB strains [14]. Superinfection allows genetic exchange to occur among *Wolbachia* strains, thereby increasing bacterial diversity [15]. Therefore, revealing the type of *Wolbachia* infections in wild *Ae. albopictus* populations is crucial for understanding the CI mechanism and *Wolbachia* evolution in *Ae. albopictus* mosquitoes.

**Figure 1:**
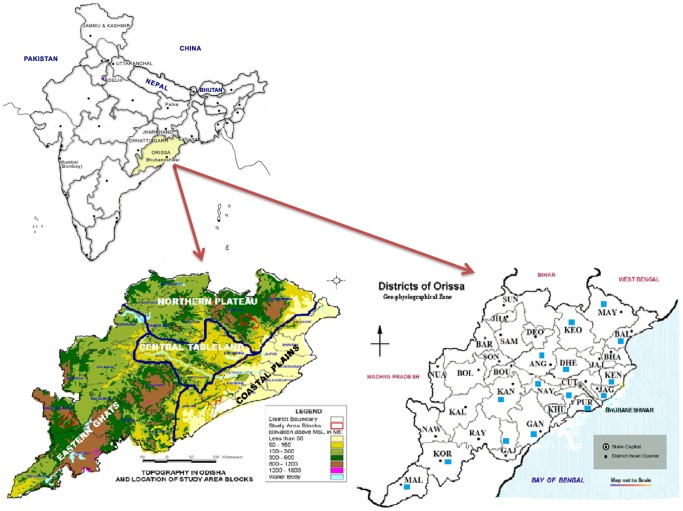
Map of Orissa showing the four physiographical regions (left view) and collection sites in blue squares (right view). Districts abbreviations are as follows: KEO- Keonjhar, MAY- Mayurbhanj, DEO-Deogarh, SUN-Sundergarh, JHA-Jharsuguda, SAM-Sambalpur, BAR-Bargarh, BAL-Balasore, BHA-Bhadrak, JAJ-Jajpur, KEN-Kendrapara, CUT-Cuttack, JAG-Jagatsinghpur, PUR-Puri, KHU-Khurdha, GAN-Ganjam, NAY-Nayagarh, ANG-Angul, DHE-Dhenkanal, SON-Sonepur, NUA-Nuapara, BOL-Bolangir, BOU-Boudh, KAN-Kandhamal, GAJ-Gajapati, RAY-Rayagada, KOR-Koraput, MAL-Malkangiri, KAL-Kalahandi, NAW-Nawrangpur.

Orissa, in the eastern part of India has been a malaria endemic region for a long period. It has different types of landscapes that are adjacent to each other: Coastal Plains (near to sea level), Central Tableland (about 150 m above sea level), Eastern Ghats and Northern Plateau (about 800 m above sea level) (Fig. 1), which act as geographical barriers. Since 2006, there have been explosive outbreaks of arboviral diseases, i.e. chikungunya and dengue in many parts of Orissa, which in turn has increased the endemicity of Orissa state for dengue and chikungunya [16]. This is supported by frequent reports of dengue and chikungunya cases from the state each year as per the Health and Family Welfare Dept, Govt. of Orissa. Previous studies have shown that *Ae. albopictus, Ae. aegypti* and *Ae. vittatus* were the main *Aedes* vectors found here [17] and implicated *Ae. albopictus* to be the major vector responsible for the arboviral outbreaks [1]–[2], [18]. Thus it is important to reveal the genetic diversity in natural populations of *Ae. albopictus* found in varied landscapes of Orissa. Furthermore, it is crucial to reveal the infection pattern of *Wolbachia* strains thriving in *Ae. albopictus* mosquitoes of Orissa, prior to exploring the potential uses of CI based strategies for control programmes. Hence the study was carried out with two objectives: Firstly to reveal the pattern of genetic differentiation and secondly to elucidate the type of *Wolbachia* infection in naturally occurring populations of *Ae. albopictus* mosquitoes collected from different topographical regions of Orissa.

## Methods

### Mosquito Sampling

Entomological survey was conducted in 15 districts belonging to 4 physiogeographical regions of Orissa (Fig. 1) by household visit for the presence of *Ae. albopictus* species from 2010–2012 (Table 1). Aquatic stages were collected from domestic containers (cement tanks, earthen pots, plastic buckets) and peri-domestic water collections (used tires, discarded small and large wastes, tree holes etc.). Larvae/pupae were sampled from different containers at each location using pipettes and dippers. Adults were collected using battery operated backpack aspirators. Collections were done in replicate in each of the surveyed areas to reduce sampling error. No specific permissions were required for these locations/activities since the field studies did not involve endangered or protected species. The collected samples were then brought to Regional Medical Research Centre insectarium, Bhubaneswar, Orissa. The collected mosquito larvae and pupae were reared to adults in water containing enamel trays, supplemented with yeast extract. The adults collected from field and those emerged from aquatic stages were examined morphologically to confirm they were *Ae. albopictus* [19]. They were then stored in individual tubes containing isopropanol at −20°C until molecular analyses.

**Table 1:** Characteristics of *Ae. albopictus* sampling sites in Orissa, Eastern India.

Geographical region	Collectionsite	Code	Geographic co-ordinates	No. of adults collected from field	No. of adults emerged from larvae/pupae	Total No. of adults
	Ganjam	Gan	19.38°N 85.07°E	24	95	119
	Khurdha	Khu	20.18°N 85.62°E	21	92	113
	Puri	Pur	19.48°N 85.49°E	17	101	118
**Coastal Plains**	Kendrapara	Ken	20.50°N 86.42°E	16	90	106
	Jagatsinghpur	Jag	20.27°N 86.17°E	18	93	111
	Balasore	Bal	21.50°N 86.90°E	19	78	97
**Central Tableland**	Angul	Ang	20.83°N 85.11°E	16	76	92
	Dhenkanal	Dhe	20.65°N85.60°E	11	65	76
	Nayagarh	Nay	20.11°N 85.01°E	14	63	77
**Northern** **Plateau**	Keonjhar	Keo	21.63°N 85.60°E	5	31	36
	Mayurbhanj	May	21.93°N 86.73°E	7	30	37
**Eastern Ghats**	Gajapati	Gaj	18.88°N 84.20°E	20	74	94
	Malkangiri	Mal	18.25°N 82.13°E	18	76	94
	Koraput	Kor	18.80°N 82.70°E	12	48	60
	Kandhamal	Kan	20.47°N 84.23°E	13	48	61
				**231**	**1060**	**1291**

### Microsatellite PCR Analysis

Total DNA was extracted from whole mosquito bodies separately as described by Coen *et al*., 1982 [20]. DNA pellets were resuspended in sterile 1X TE buffer and stored at −20°C. Genetic polymorphism was assessed at seven microsatellite markers: AealbA9, AealbB6, AealbB52, AealbB51, AealbD2, AealbF3 [21], and AEDC [22] (Table S1). DNA was amplified in an ABI thermal cycler (ABI Biosystems, USA) in 20-μl reaction mixes containing 50 ng of template DNA, 2.5 μl of 10 X reaction buffer (Sigma, USA), 0.4 mM MgCl_2_ (for AealbB6 and AealbD2), 0.8 μl of 10 mM dNTP mix (Sigma, USA), 25 pmol of each primer, 1 U of Taq polymerase (Sigma, USA) and nuclease free water. The 5′ end of each forward primer was labeled with a fluorescent dye (FAM, HEX or VIC) (Sigma, USA). Cycling conditions were as follows: 5 min at 94°C, followed by 35 cycles of 94°C for 40 secs, annealing temperature (T_A_) (Table S1) for 40 secs, 72°C for 1 min, and 72°C for 30 mins and finally stored at 4°C. 3 μl of each amplified product was diluted in water (1/50 to 1/100, according to the sensitivity of each loci) and aliquoted in 0.2 ml eppendorff tubes. 0.4 μl of GS 500 Liz internal size standard™ (allele size marker) (Applied Biosystems, USA) and 9.6 μl of Hi-Di-formamide was added to 10 μl of each diluted amplification product, to obtain a total volume of 20 μl. The mixture was pre-heated at 94°C for 3 minutes, followed by microsatellite analysis in an ABI PrismTM 3130 XL Genetic Analyzer (Applied Biosystems, USA) as per the manufacturer’s instructions. Microsatellite alleles were scored with GeneMapper software (Applied Biosystems, USA).

### Genetic Diversity Analysis

Genetic diversity within *Ae. albopictus* populations was assessed at each locus by estimating allele frequencies, number of alleles A, allele richness Rs, inbreeding coefficient F_IS_, expected heterozygosity He, and observed heterozygosity Ho [23], using the software FSTAT 2.9.3.2 [24]. The frequency of null alleles (r) within each population was determined using the Brookfield 2 estimate [25], and the allele and genotype frequencies were then adjusted accordingly in MICRO-CHECKER 2.2.3 [26]. The null allele-adjusted dataset was compared to the original dataset to examine the impact of null alleles on the estimations of genetic differentiation. Genotypic frequencies were tested against Hardy-Weinberg equilibrium (HWE) at each locus in the pooled populations as well as in each mosquito population. Statistical significance was assessed by the exact probability test available in GENEPOP 3.2 [27]. Linkage disequilibrium between the microsatellite loci was tested by Fisher exact tests on contingency tables, also available in GENEPOP 3.2.

Population genetic differentiation was measured by the fixation index, F_ST_ [28] and statistical significance was assessed by the exact test of genotypic differentiation available in FSTAT 2.9.3.2. Analysis of molecular variation (AMOVA) was undertaken in Arlequin v3.11 [29] using pairwise F_ST_ as the distance measure, with 10,000 randomizations and missing data for loci set at 10%. The model for analysis partitioned variation among geographical regions, among and within populations. Since geographical isolation is often considered as the main driving force for population differentiation, the logarithm of geographical distances [Fst/(1-Fst)] was calculated between sampling sites by employing a Mantel test [30] as implemented in the web service Isolation by Distance [31] using 10,000 randomizations. Significance level of multiple testing was corrected using sequential Bonferroni’s procedures [32]. Chord distances between each pair of populations were calculated in GENETIX 4.05 [33] and a neighbor-joining tree was generated using MEGA 5.01 [34] employing distance-based cluster analyses.

Since demographic instability such as recent population bottleneck and/or expansion might bias genetic differentiation estimates to a significant extent [35], [36], heterozygosity tests [37] were employed to detect deviations from mutation-drift equilibrium (MDE) using Bottleneck V.1.2.0.2. These tests compare two estimates of expected heterozygosity, one based on allele frequencies (He), assuming Hardy-Weinberg proportions, and another based on the observed number of alleles and sample size (Heq), assuming MDE. At MDE, both estimates should be similar in the majority of loci analyzed (i.e. He = Heq, it means heterozygote deficit = heterozygote excess). If a population experiences a bottleneck, rare alleles will be rapidly lost and therefore Heq will decrease faster than He (i.e. He>Heq). This apparent excess of heterozygosity in a significant number of loci is an indicator of a bottleneck, whereas the converse (i.e. He<Heq) may indicate a population expansion. Estimates of *H*eq were computed using two mutation models for microsatellites evolution: Two- Phased Mutation model (TPM) [38] with fractions of multistep mutations set to 30%, 20% and 10%, and the Stepwise Mutation Model (SMM) [39]. Empirical data suggest that TPM model is more appropriate than SMM for microsatellite loci. Statistical significance of deviations from MDE was assessed by estimating p value of the loci showing significant heterozygote deficiency using Wilcoxon signed rank tests available in Graphpad Prism 6 software.

### Cluster Analysis

A Bayesian approach was used to deduce the number of clusters (K) in the data set without prior information of the sampling locations in STRUCTURE 2.3 [40]. A model where the allele frequencies were correlated within populations was assumed (λ was set at 1, the default value) and the software was run with the option of admixture, allowing for some mixed ancestry within individuals, and α was allowed to vary. Fifteen independent runs were performed for each assumed number of populations (K = 1 to 15), with a burn-in period of 100,000 iterations and 500,000 replications. The method of Evanno [41] was used to determine the most likely number of clusters. This approach uses an ad hoc quantity, ΔK, based on the second order rate of change of the likelihood function between successive values of K. Additionally, a factorial correspondence analysis (FCA) was performed to assess the clustering pattern by analyzing the allele frequencies and genetic distances among the specimens and a 3D plot of the FCA was generated using XLSTAT program.

### *Wolbachia* Genotyping

The quality of DNA extracted from individual mosquitoes collected from different regions of Orissa (Fig. 1) was assessed by performing species specific PCR using 18S ribosomal DNA [17]. All the specimens were screened initially for *Wolbachia* infection by PCR using *wsp* gene, (81F, 5′-TGGTCCAATAAGTGATGAAGAAAC-3′ and 691R, 5′-AAAAATTAAACGCTACTCCA-3′), which amplified 615 bp product. This served as positive control for *Wolbachia* genotyping. Then, *Wolbachia* genotyping in *Ae. albopictus* samples was performed by a multiplex PCR using *Wolbachia*-specific primers, 328F/691R for wAlb A and 183F/691R for wAlb B (328F, 5′-CCA GCA GAT ACT ATT GCG-3′; 183F,5′–AAG GAA CCG AAG TTC ATG-3′; 691R, 5′-AAA AAT TAA ACG CTA CTC CA-3′) [12]. The PCR reaction mixture comprised 0.25 mM dNTPs, 2.5 mM MgCl_2_, 0.2 mM primers and 1 U Taq DNA polymerase (Sigma) with the following thermal cycler conditions: 95°C for 5 mins, followed by 35 cycles of 95°C for 1 min, 54.4°C for 1 min and 72°C for 2 min, followed by 72°C for 10 min. Out of the *Wolbachia* positive *Ae. albopictus* specimens, confirmation of *Wolbachia* strains in 5 mosquito samples representing the four physiographical regions was accomplished through sequencing and phylogenetic analyses.

### Sequencing and Phylogenetic Analyses

The PCR products were visualized on a 1.5% low melting point agarose gel at 300 nm under UV transilluminator. The PCR products of wAlb A (379 bp) and wAlb B (501 bp) using 328F/691R and 183F/691R primers of the *wsp* gene were excised from gel, purified using QIAquick Spin Column (Qiagen, Hilden, Germany) and sequencing PCR done. For each sequencing reaction, 50 ng of purified PCR product was mixed with a reaction mixture containing 2.5 X sequencing buffer, 5 X big dye terminator and 20 μM of either forward/reverse primers of *w*Alb A and *w*Alb B genes in two separate sets of reaction. Cycle sequencing parameters used were: 96°C for 1 min followed by 25 cycles of 96°C for 10 secs, 50°C for 5 sec, and 60°C for 4 min. The PCR product was purified by EDTA/ethanol precipitation. The dried pellet was resuspended in 10 μl of Hi-Di-formamide, and sequenced in a 16 capillary (90 cm) automated DNA sequencer (Applied Biosystems, Foster City, Ca, USA) using performance optimized polymer 7 following the manufacturer’s instructions. The nucleotide sequences obtained after sequencing were edited and analyzed by the Sequencing analysis software (Applied Biosystems, Foster City, Ca, USA). The *wsp* gene sequences generated have been deposited in GenBank with accession nos. **JX475999–JX476007** (wAlbA isolates), **JX629463–JX629467** (wAlbB isolates). Multiple sequence alignments and phylogenetic analysis were performed using partial *wsp* gene sequences of the mosquito isolates obtained from Orissa and other regions in Mega 5 software (Arizona State University, Tempe, AZ, USA) [34]. All gap containing codons were deleted and homogenous patterns among the groups and uniform rates among sites were assumed. The best fit model of nucleotide substitution was selected by using Akaike Information Criterion (AIC) as implemented in Modeltest version 3.7. The phylogenetic tree was constructed by using the maximum-likelihood method using Tamura Nei model of Mega 5 software. The robustness of each node was estimated using 1000 bootstrap replications under the Nearest-Neighbor Interchange procedure, with input genetic distance determined under the maximum-likelihood substitution model. Additionally, a WSP typing system, based on the amino acid sequences encoded by the four hypervariable regions (HVRs) of the *wsp* gene was employed to compare the *wsp* sequences obtained from *Wolbachia* strains thriving in natural populations of *Ae. albopictus* of Orissa with the HVR profiles of the *wsp* gene available in PubMLST database [42].

### Statistical Analyses

*Wolbachia* infection/superinfection rates were estimated for *Ae. albopictus* mosquitoes in each of the surveyed districts belonging to the four topographical regions of Orissa. Comparison was done between *Wolbachia* superinfection in *Ae. albopictus* mosquitoes of the 4 regions using non parametric Kruskal Wallis ANOVA, which calculated the two-tailed p values using the Graphpad Prism (version 6) software. A p value <0.05 was considered to be statistically significant.

## Results

### Microsatellite Loci Variability

Genotypes at 7 microsatellites loci were assessed in a total of 1291 *Ae. albopictus* mosquitoes collected from 15 districts of 4 different physiographical regions of Orissa. Polymorphism at the 7 microsatellite loci varied, with number of alleles as 18 (AealbA9), 11 (AaealbD2 and AealbB6), 7 (AealbF3), 6 (AEDC), 5 (AealbB51) and 4 (AealbB52) respectively (Table 2). The average number of alleles per locus was in a range between 4 (AealbB52) to 9 (AealbA9). The minimum mean number of alleles of all loci was in KEO population (3.2), and the maximum in GAN population (6.8) (Table 2). Mean allelic richness (Rs) across populations ranged from 2.23 at locus AealbB52 to 7.87 at locus AealbA9. The average observed heterozygosity (Ho) across all samples ranged from 0.163 (AealbB52) to 0.685 (AealbA9), the minimum Ho was in KEO (0.169) and the maximum Ho in GAN (0.485). To assess the impact of null alleles on population genetic analyses, these analyses were performed both before and after the dataset were adjusted for estimated null allele frequencies. The effect of this treatment was minimal and did not significantly change the degree or statistical significance of the estimated parameters. Departure from HWE (P<0.05 after correction for multiple testing) associated with significant heterozygote frequency deficits (positive value of F_IS_) were detected in 2 of 7 (28.5%) loci when all samples were pooled and considered as a single gene pool, suggesting population substructure. At the population level, 8 out of 105 (7.6%) comparisons did not conform to Hardy-Weinberg expectations after sequential Bonferroni correction, and the deviations were associated with positive inbreeding coefficient (F_IS_), reflecting heterozygosity deficits (Table 2). Significant deviation from HWE varied across loci in a population-dependent manner. NAY, ANG and GAJ populations had the highest number of loci in departure from HWE (2 of 7), while the JAG and MAL populations had the least (1 of 7). The remaining populations had no loci in departure from HWE. In all samples, some specimen failed to amplify at one locus while succeeded at the remaining loci, suggesting the presence of null alleles. Estimates of the frequency of null alleles are given in Table 2.

**Table 2:** Summary of microsatellite variation at 7 loci for Ae. albopictus in Orissa, India.

Locus		Gan	Khu	Pur	Ken	Jag	Bal	Nay	Ang	Dhe	Keo	May	Gaj	Kan	Kor	Mal	All samples
		119	113	118	106	111	97	77	92	76	36	37	94	61	60	94	1291
**AealbA9**	A	11	9	10	10	11	9	8	9	8	5	5	9	9	7	9	18
	Rs	9.528	8.027	9.172	9.169	9.692	8.337	7.207	8.346	7.391	4.856	4.899	8.167	8.383	6.907	7.987	7.87
	He	0.877	0.812	0.829	0.801	0.818	0.777	0.649	0.786	0.692	0.378	0.377	0.744	0.788	0.567	0.678	0.704
	Ho	0.881	0.836	0.841	0.82	0.83	0.736	0.624	0.797	0.673	0.358	0.367	0.733	0.729	0.523	0.621	0.685
	r	–	–	–	–	–	0.001	0.003	–	0.003	0.011	0.012	0.004	0.004	0.002	0.003	0.003
	F_IS_	−0.16	−0.11	−0.18	−0.07	−0.03	0.003	0.022	0.015	0.034	0.099	0.046	0.065	0.073	0.105	0.112	0.014
**AealbD2**	A	8	6	7	7	6	5	3	6	4	3	3	5	4	4	5	11
	Rs	5.616	5.143	5.482	5.737	5.345	4.093	1.547	4.498	2.675	1.258	1.062	2.155	2.099	2.345	3.897	3.587
	He	0.495	0.429	0.454	0.436	0.424	0.32	0.118	0.358	0.129	0.085	0.08	0.14	0.144	0.132	0.201	0.248
	Ho	0.513	0.447	0.5	0.476	0.446	0.393	0.033	0.333	0.182	0.101	0.085	0.134	0.098	0.112	0.212	0.273
	r	–	–	–	–	–	–	0.067	0.009	–	–	–	0.006	0.01	0.012	–	0.003
	F_IS_	−0.17	−0.16	−0.14	0.05	−0.1	−0.14	**0.161**	0.113	0.06	−0.07	0.048	0.037	0.127	0.036	0.004	0.002
**AealbB51**	A	4	4	4	3	4	3	4	4	3	3	3	4	4	3	4	5
	Rs	3.423	3.231	3.133	2.171	3.332	2.422	3.028	3.367	2.853	1.383	1.282	2.116	2.372	2.087	2.987	2.64
	He	0.288	0.219	0.256	0.161	0.257	0.187	0.286	0.281	0.244	0.108	0.085	0.125	0.166	0.176	0.211	0.218
	Ho	0.284	0.204	0.131	0.157	0.251	0.111	0.157	0.285	0.202	0.087	0.078	0.116	0.148	0.17	0.154	0.171
	r	0.002	0.003	0.058	–	0.019	0.045	0.074	–	0.037	0.003	0.006	0.011	0.019	0.002	0.054	0.029
	F_IS_	0.005	0.013	0.047	0.009	0.033	0.131	**0.167**	−0.03	0.131	0.017	0.078	0.096	0.048	0.035	0.093	0.071
**AealbB6**	A	8	6	7	7	8	5	4	6	5	4	4	6	4	5	5	11
	Rs	6.286	5.275	6.072	6.139	7.132	4.057	3.067	5.346	4.219	2.568	2.989	5.117	3.083	3.917	4.027	4.67
	He	0.617	0.522	0.539	0.511	0.638	0.347	0.309	0.485	0.422	0.208	0.287	0.374	0.288	0.317	0.368	0.412
	Ho	0.511	0.436	0.481	0.47	0.434	0.312	0.304	0.398	0.373	0.178	0.267	0.243	0.279	0.217	0.151	0.324
	r	0.028	0.052	0.047	0.046	0.143	0.021	0.009	0.065	0.087	0.025	0.022	0.104	0.004	0.092	0.133	0.067
	F_IS_	0.065	0.076	0.086	0.074	**0.223**	0.093	0.032	0.085	0.132	0.089	0.076	**0.165**	0.033	0.115	**0.212**	0.135
**AealbF3**	A	5	5	4	5	5	5	3	4	4	3	3	5	4	4	4	7
	Rs	4.165	4.134	2.982	4.137	4.175	3.938	1.547	3.478	2.985	2.238	2.682	4.125	3.129	3.345	3.277	3.355
	He	0.375	0.379	0.254	0.364	0.314	0.313	0.083	0.308	0.219	0.146	0.168	0.315	0.197	0.233	0.273	0.262
	Ho	0.387	0.417	0.301	0.374	0.326	0.393	0.073	0.243	0.187	0.133	0.125	0.278	0.198	0.212	0.257	0.293
	r	–	–	–	–	–	–	0.021	0.059	0.065	0.023	0.031	0.056	–	0.022	0.023	0.002
	F_IS_	−0.04	−0.06	−0.14	0.005	0.01	−0.14	0.037	0.073	0.086	0.047	0.078	0.117	0.013	0.066	0.067	0.014
**AEDC**	A	5	3	4	5	4	4	3	4	4	3	3	2	2	3	3	6
	Rs	4.138	2.234	3.124	4.076	3.182	2.982	2.918	3.135	3.152	2.833	2.182	1.116	1.372	2.817	2.187	2.742
	He	0.317	0.194	0.265	0.292	0.234	0.201	0.236	0.214	0.221	0.188	0.152	0.095	0.106	0.171	0.123	0.2
	Ho	0.344	0.189	0.213	0.287	0.255	0.213	0.222	0.165	0.202	0.134	0.148	0.093	0.088	0.17	0.115	0.189
	r	–	0.007	0.011	0.005	–	–	0.056	0.068	0.037	0.021	0.004	0.002	0.019	0.002	0.024	0.017
	F_IS_	−0	0.073	0.085	0.051	0.002	−0.02	0.085	**0.177**	0.034	0.092	0.038	0.026	0.128	0.024	0.103	0.066
**AealbB52**	A	4	4	4	3	4	3	4	3	3	2	2	4	3	3	4	4
	Rs	3.548	3.234	3.276	2.434	3.334	3.334	3.018	2.482	2.853	1.383	1.282	3.295	2.372	2.087	2.987	2.232
	He	0.273	0.244	0.216	0.199	0.295	0.295	0.286	0.19	0.244	0.088	0.085	0.288	0.186	0.176	0.211	0.204
	Ho	0.229	0.188	0.257	0.184	0.113	0.113	0.282	0.063	0.202	0.071	0.068	0.092	0.148	0.151	0.144	0.163
	r	0.023	0.008	0.003	0.002	0.031	0.051	0.002	0.085	0.037	0.002	0.016	0.071	0.019	0.012	0.054	0.038
	F_IS_	0.057	0.081	0.018	0.023	0.076	0.085	0.011	**0.169**	0.101	0.016	0.078	**0.172**	0.068	0.034	0.103	0.094
**All loci**	A	6.81	5.3	6	6.3	6.33	5.16	4.16	5.5	4.6	3.23	3.33	5	4.33	4.33	5	9.5
	Rs	5.506	4.541	5.027	5.422	5.48	4.314	3.217	4.683	3.879	2.522	2.516	3.799	3.406	3.569	4.06	4.144
	He	0.491	0.422	0.439	0.436	0.454	0.358	0.264	0.406	0.321	0.182	0.191	0.298	0.284	0.266	0.309	0.34
	Ho	0.485	0.403	0.401	0.439	0.415	0.36	0.261	0.35	0.303	0.169	0.178	0.266	0.24	0.234	0.25	0.321
	r	0.009	0.02	0.024	0.013	0.03	0.009	0.016	0.044	0.038	0.013	0.012	0.031	0.024	0.022	0.039	0.02
	F_IS_	−0.03	0.006	−0.02	0.033	0.056	−0.01	0.082	0.105	0.097	0.048	0.077	0.102	0.087	0.08	0.1	0.053

N, sample size; A, number of alleles; Rs, allelic richness; He, expected heterozygosity; Ho, observed heterozygosity; r, estimated frequency of null alleles; F_IS_, inbreeding coefficient; All loci/samples, mean values over loci or populations; –, no significant heterozygote deficiency;Values in bold highlight significant deficit in heterozygote (P<0.01 after Bonferroni correction).

Fisher’s exact tests were conducted for linkage disequilibrium (LD) within each of the 15 collections. Out of 105 comparisons only three pairs (2.81%) were at LD (P<0.05). The three pairs were detected in NAY (AealbD2/AealbB51), ANG (AEDC/AealbB52) and GAJ (AealbB6/AealbB52) populations respectively. No pair of loci appeared in LD in more than one population, suggesting genetic independence between loci. When the test was performed in the pooled populations, no pairs of loci out of 10 possible combinations showed significant P values (P.0.05).

### Genetic Differentiation

**Figure 2:**
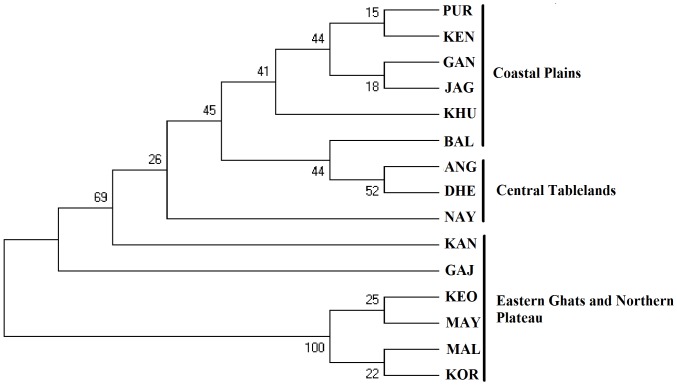
Dendrogram based on chord distance clustering by NJ method using Mega 5.01. The genetic relationship among 15 *Ae. albopictus* populations sampled in Orissa is shown.

Overall genetic differentiation between populations was moderate and statistically significant (F_ST_ = 0.088, P = 0.001). Pairwise analysis of populations indicated significant genetic differentiation for most sample pairs (91/105), associated with F_ST_ estimates ranging from 0.009 (KEO-KOR) to 0.223 (GAN/KEO). The F_ST_ values were non-significant for most of the Northern Plateau and Eastern Ghats population comparisons, such as KEO/MAY, KOR/MAL, etc. (Table 3) which indicated low genetic differentiation between these populations. For all other population comparisons, the values were significant suggesting high genetic differentiation. The average genetic diversity among the populations of the Coastal Plains was low (F_ST_ = 0.052), indicating high genetic similarity between them. However, high and statistically significant genetic differentiation (average F_ST = _0.144) was recorded when comparison was performed between coastal plain populations and other populations. Genetic divergence was maximum when comparison was performed between any coastal plain population with Northern Ghats populations (Table 3). Pairwise comparison of Central Tablelands populations (except ANG population) with other populations did not reveal significant genetic differentiation. The NJ tree constructed with pairwise chord distances and bootstrap values supporting node clearly indicated that the Coastal Plains populations were clustered together and were genetically distinct from all the other populations, with a single exception, BAL, which remained isolated (Fig. 2). Similarly the KEO, MAY, KOR and MAL populations formed a distinct group, supported by high bootstrap values, thereby indicating high genetic resemblance among these populations, although they represented different geographical regions. AMOVA analysis indicated significant differences among regions as well as among populations within regions. Differences among regions accounted for 29.65% (p<0.0001) of the observed genetic variation, whereas the variance between the populations was 14.09% (p<0.0001) and within populations was 56.26% (p<0.001) (Table S2). These results showed great interpopulation genetic diversity in the *Ae. albopictus* populations of Orissa.

**Table 3:** Matrix of pairwise estimates of F_ST_ among *Ae. albopictus* populations from Orissa, India.

Regions	Population	Gan	Khu	Pur	Ken	Jag	Bal	Ang	Dhe	Nay	Keo	May	Gaj	Kor	Mal	Kan
**Coastal Plains**	**Gan**															
	**Khu**	**0.041**														
	**Pur**	**0.031**	**0.035**													
	**Ken**	**0.075**	**0.046**	**0.014**												
	**Jag**	**0.021**	**0.043**	**0.026**	**0.015**											
	**Bal**	**0.093**	**0.058**	**0.072**	**0.051**	**0.058**										
**Central Tablelands**	**Ang**	**0.096**	**0.075**	**0.082**	**0.073**	**0.065**	**0.045**									
	**Dhe**	**0.106**	**0.112**	**0.129**	**0.084**	**0.072**	**0.062**	**0.053**								
	**Nay**	**0.119**	**0.126**	**0.113**	**0.117**	**0.12**	**0.126**	**0.117**	0.097							
**Northern Plateau**	**Keo**	**0.223**	**0.143**	**0.191**	**0.164**	**0.173**	**0.133**	**0.124**	**0.119**	**0.121**						
	**May**	**0.177**	**0.134**	**0.201**	**0.115**	**0.123**	**0.124**	**0.115**	**0.117**	**0.112**	0.009					
**Eastern Ghats**	**Gaj**	**0.101**	**0.126**	**0.125**	**0.117**	**0.126**	**0.146**	**0.147**	**0.123**	**0.114**	0.067	0.066				
	**Kor**	**0.158**	**0.129**	**0.162**	**0.125**	**0.119**	**0.183**	**0.125**	**0.127**	**0.122**	0.046	0.057	0.059			
	**Mal**	**0.125**	**0.121**	**0.128**	**0.121**	**0.129**	**0.18**	**0.132**	**0.125**	**0.139**	0.047	0.053	0.055	0.023		
	**Kan**	**0.149**	**0.117**	**0.115**	**0.126**	**0.124**	**0.177**	**0.126**	**0.136**	**0.123**	**0.088**	**0.085**	0.026	0.068	0.069	

In bold, significant P values (P<0.01) after correction for multiple tests.

**Figure 3:**
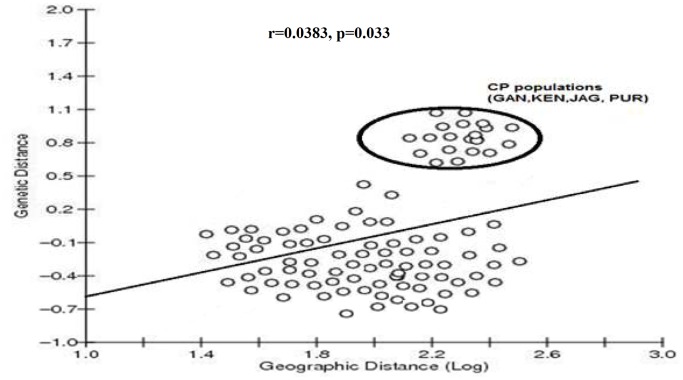
Correlation between genetic distance (Fst/(1-Fst) and logarithm of geographic distance of *Ae. albopictus* populations from Orissa. Coastal plains populations (CP) showed some amount of positive correlation when compared with other populations.

In most populations, no statistically significant positive correlation was detected between genetic differentiation and geographic distance. However some evidence of positive correlation was observed when GAN, KEN, JAG and PUR of Coastal Plains were compared with other populations (r = 0.383, p = 0.033) (Fig. 3). Furthermore, the topology of the tree shown in Fig. 2 suggested that the pattern of genetic differentiation was not shaped by the geographic distance between sampling sites. The results suggest that geographic distance does not significantly contribute to the genetic differentiation observed in *Ae. albopictus* populations of Orissa.

### Bottleneck Analysis

Heterozygosity tests were performed to explore the demographic stability in *Ae. albopictus* populations and compliance to MDE. No signature of a recent bottleneck event (population reduction/expansion) was detected by TPM in any populations. Significant deviations (P<0.01) from MDE (He<Heq) (in 3 loci showing significant heterozygote deficiency) were found in ANG and GAJ populations under the SMM (Table 4), which may suggest population expansion.

**Table 4:** Heterozygosity tests in *Ae. albopictus* populations from Orissa, India.

Regions	Populations	TPM			SMM
		70%	80%	90%	
**Coastal Plains**	GAN	0	0	1	1
	PUR	1	1	1	1
	KHU	0	1	1	1
	KEN	0	1	1	1
	JAG	0	1	1	1
	BAL	0	0	0	1
**Central Tablelands**	NAY	1	2	2	2* (AealbD2, AealbB51)
	ANG	1	2	2	3*** (AealbB6, AealbB51, AealbB52)
	DHE	0	0	1	1
**Northern Plateau**	KEO	0	0	0	0
	MAY	0	0	0	0
**Eastern Ghats**	GAJ	1	1	2	3** (AealbB6, AEDC, AealbB52)
	KAN	0	0	1	1
	KOR	0	0	1	1
	MAL	1	1	2	2

TPM: Two-Phase mutation Model with a % single step mutations, SMM: Stepwise Mutation Model;

*P<0.05, **P<0.01 and ***P<0.001 (two-tailed Wilcoxon signed-rank test P-values for deviation from MDE after correction for multiple testing). The number of microsatellite loci showing heterozygote deficiency (He< Heq) out of 7 loci is given.

### Cluster Analysis

**Figure 4:**
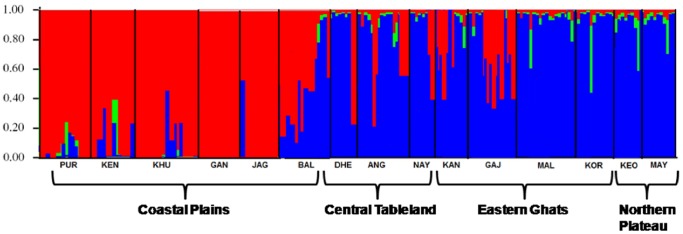
Bayesian cluster analysis using STUCTURE. The data set showing the most likely clusters (K = 3), where each color corresponds to a suggested cluster and each individual is represented by a vertical bar. The X-axis corresponds to population codes. The Y-axis presents the probability of assignment of an individual to each cluster.

**Figure 5:**
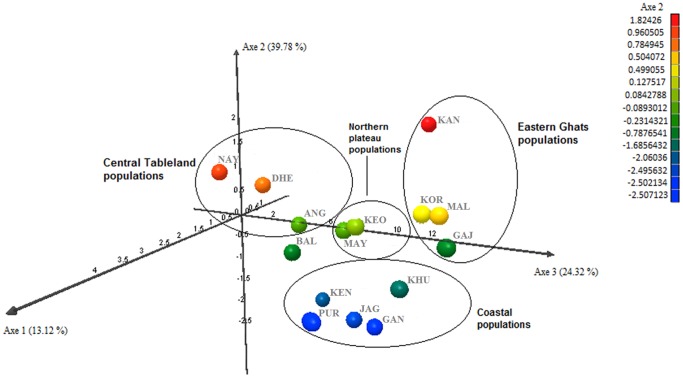
3D plot of the FCA analysis showing most Coastal plains populations (encircled) to be genetically isolated from other populations.

The Bayesian cluster analysis divided the populations into three clusters (posterior probability of Bayesian clustering Ln(D) likelihood score optimal for k = 3 clusters) (Fig. 4). More than 75% of Coastal Plains populations belonging to GAN, KHU, PUR, KEN and JAG comprised of cluster I and more than 70% of populations belonging to DHE, NAY, MAY, KEO, KOR and MAL comprised of cluster II. Cluster III was a rare cluster found in some populations, mainly in KEO, MAY, KOR and MAL (>10%). All the 3 clusters were found in most populations (except GAN population), may be due to some degree of admixing between the populations. In different localities, there were different proportions of individuals from different gene pools (Table 5). For example, in the PUR population, 81.2% of individuals were assigned to cluster I, 10.5% to cluster II and 8.3% assigned to cluster III. The FCA analyses supported the results of the STRUCTURE analysis, which showed that most Coastal Plains populations were clustered together and relatively isolated from other populations (Fig. 5). Most Central Tablelands, Eastern Ghats and Northern Plateau populations were evenly distributed in the 3D plot. However KOR/MAL (Northern Plateau) and KEO/MAY (Eastern Ghats) were found in close proximity suggesting them to be genetically related.

**Table 5:** Probability of assignment of individuals to each population cluster.

Regions	Populations	1	2	3	No. of loci in departure from HWE
**Coastal Plains**	PUR	0.812	0.105	0.083	1
	KEN	0.778	0.124	0.098	1
	KHU	0.916	0.079	0.005	1
	GAN	1	0	0	0
	JAG	0.976	0.024	0	1
	BAL	0.472	0.506	0.022	0
**Central Tablelands**	DHE	0.204	0.788	0.008	2
	ANG	0.282	0.69	0.028	2
	NAY	0.156	0.837	0.007	2
**Northern Plateau**	KEO	0.035	0.842	0.123	0
	MAY	0.081	0.812	0.107	1
**Eastern Ghats**	GAJ	0.401	0.585	0.014	2
	KOR	0.073	0.806	0.121	1
	MAL	0.053	0.821	0.126	1
	KAN	0.254	0.679	0.067	1

### *Wolbachia* Genotyping

**Figure 6:**
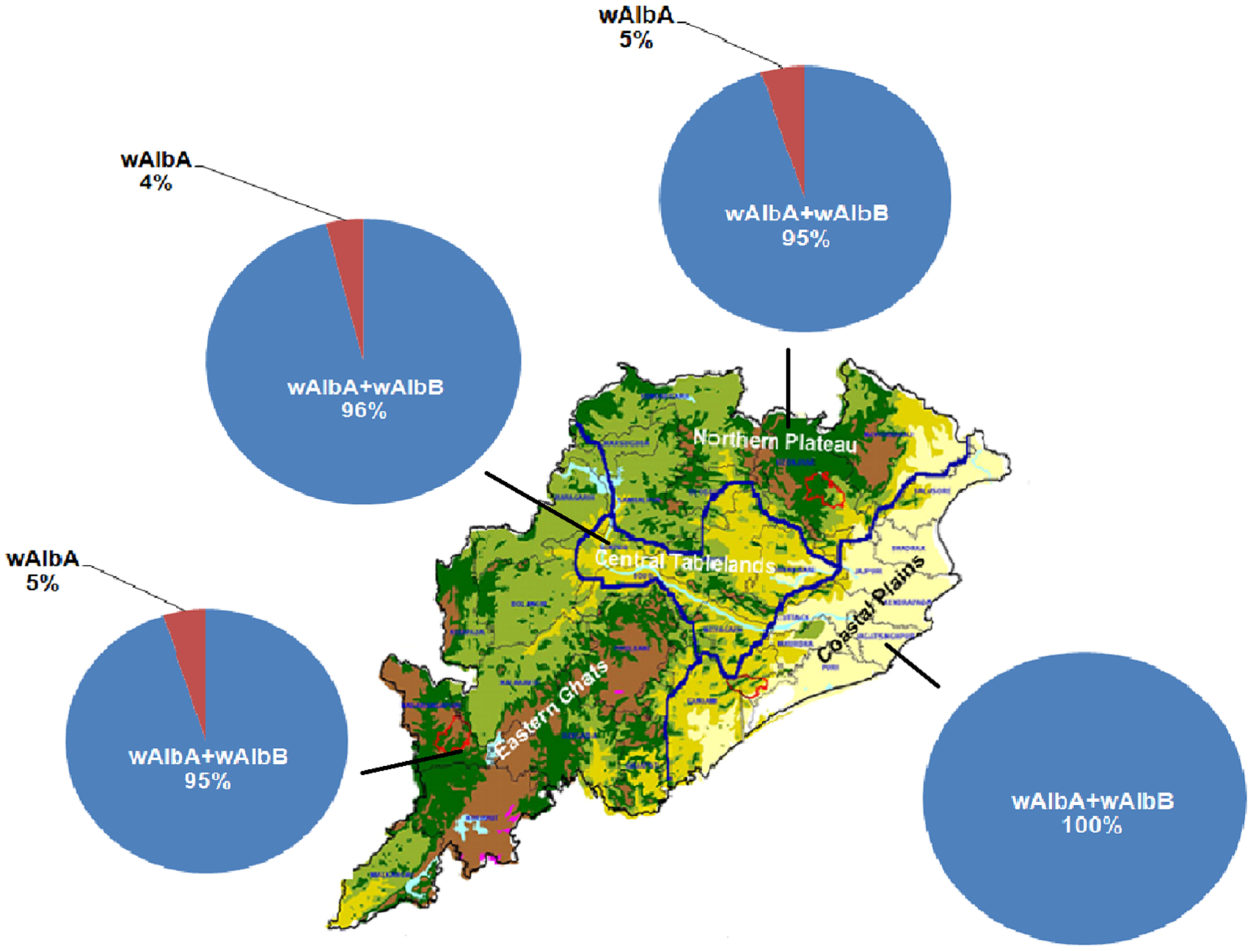
*Wolbachia* infection/superinfection rates in *Ae. albopictus* populations across the four physiographical regions of Orissa.

Out of 1291 (689 males and 602 females) *Ae.albopictus* collected from different regions of Orissa, 1281 (681 males and 600 females) (99.2%) mosquitoes showed band at 615 bp (*wsp*), thereby indicating to be infected with *Wolbachia*. Multiplex PCR analysis of 1281 individuals using group specific primers depicted that 41 (3.2%) specimens exhibited band at 379 bp, thus infected with wAlbA strain and 1240 (96.8%) specimens exhibited bands at 379 bp and 501 bp, thereby indicating to be super-infected with wAlbA and wAlbB strains. Furthermore, 100% *Wolbachia* superinfection (wAlbA+wAlbB) was detected in *Ae. albopictus* mosquitoes collected from the Coastal Plains. Northern Plateau, Eastern Ghats and Central Tablelands populations recorded super-infection rates of 95%, 96% and 96% in the mosquitoes (Fig. 6). Statistical analyses revealed that *Wolbachia* superinfection in the coastal plains was significantly high in comparison to other regions (Kruskal Wallis statistic, K: 11.7, p = 0.0075). Of the *Wolbachia* infected males, 95.7% (652/681) were super infected with wAlbA and wAlbB strains and 4.3% (29/681) infected with only wAlbA. Superinfection of wAlbA and wAlbB strains was detected in 98% females (588/600), while only wAlbA infection was observed in 2% (12/600) females. We did not detect any mosquito specimen to be singly infected with wAlbB.

**Figure 7:**
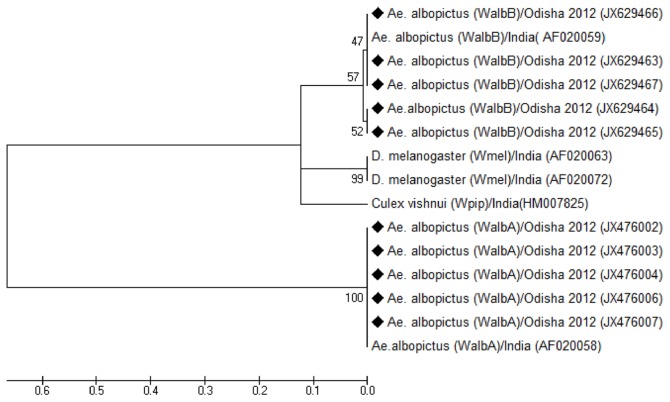
Phylogenetic analysis of *Wolbachia.* Phylogenetic tree based on *wsp* sequence of *Wolbachia*, constructed by Neighbour-Joining algorithm confirmed the circulation of wAlbA and wAlbB in *Ae. albopictus* mosquitoes of Orissa. Numbers on the nodes indicates bootstrap percentage of 1000 replicates. Solid diamonds represent the mosquito isolates sequenced in this study.

Phylogenetic analyses of *wsp* gene sequences of *Wolbachia* extracted from *Ae. albopictus* mosquitoes of Orissa and other regions confirmed the circulation of wAlbA and wAlbB strains. High genetic divergence (GD: 0.234) was observed between the supergroup A and B that resulted in population subdivision, which was also supported by high bootstrap values at each node. There was low genetic variability in the populations within group A (GD: 0.0043) as well as populations within group B (GD: 0.0042). The phylogenetic tree confirmed the co-infection of wAlbA and wAlbB in *Ae. albopictus* mosquitoes collected from different regions of Orissa (Fig. 7). Furthermore, comparison of *Wolbachia* sequences with the HVR profiles through PubMLST revealed that Orissa wAlbA strains shared the HVR1∶1, HVR2∶1, HVR3∶1 and HVR4∶1 alleles, whereas the wAlbB strains shared the HVR1∶10, HVR2∶82, HVR3∶10 and HVR4∶84 alleles, which further verified the circulation of wAlbA and wAlbB in *Ae. albopictus*, thus supporting the results of the phylogenetic analyses.

## Discussion

Understanding the patterns of gene flow and population genetic structure of invasive vectors like *Ae. albopictus* is important for designing and interpreting the outcome of area-wide elimination strategies. This is the first study on the genetic differentiation alongwith revealing the type of *Wolbachia* infection in naturally occurring populations of *Ae. albopictus* in an emerging arboviral endemic state of India. The seven microsatellites used in this study for assessing the genetic diversity of *Ae. albopictus* populations were highly polymorphic (except AealbB52), with small number of deviations from HWE and thus were useful for exploring *Ae. albopictus* population genetic structure. The set of microsatellite makers used here had already been successfully tested on habitat structuring *Ae. albopictus* in Reunion islands [43]. The number of alleles detected in our study of a larger number of field populations was significantly higher at all loci, except for AaelB52 (4). Deviations from HWE observed in some samples could be attributed to the presence of null alleles or Wahlund effect. The heterozygote deficits at several loci detected in this study was possibly due to the presence of null alleles since there were some specimens that failed to amplify some alleles at certain loci, but efficiently amplified at other loci. Another cause for the reduction in heterozygotes may be due to the subpopulations in the sample or Wahlund effect. In fact, the Bayesian cluster analysis revealed three genetic clusters across the locations sampled (Fig. 4), and the three genetic clusters coexisted in most of the populations (Table 5). Thus, a part of the heterozygote deficits detected in our samples could be the result of the Wahlund effect.

The pattern of genetic differentiation estimated from the microsatellite data indicated a significant level of genetic structuring among *Ae. albopictus* populations in Orissa. No obvious correlation between genetic and geographic distances was found, suggesting that the genetic structure has been shaped by additional biotic or abiotic factors. However, some evidence of correlation (r = 0.383, p = 0.033) was observed when the coastal plain populations were taken as a unit and compared with other populations. Such findings indicated that the coastal plain populations were genetically more differentiated than the other populations. This was also supported by high genetic differentiation (F_ST_ >0.125) when comparison was done between Coastal Plains populations with other populations (Table 3). Furthermore, AMOVA analyses indicated great interpopulation genetic diversity among the populations between the regions (mainly between Coastal Plains and other regions). However genetic differentiation among the Coastal Plains populations was low, thus indicating high genetic resemblance among them. The Bayesian cluster analysis depicted the existence of 3 genetic clusters in the *Ae. albopictus* populations and separated the Coastal Plains populations (except BAL) as unique genetic cluster, thereby rendering to be genetically divergent from other populations. Such type of genetic uniqueness of the Coastal Plains populations can be attributed to the ecological landscapes that form geographical barriers and separate it from other populations and thus act as a source for genetic diversity in *Ae. albopictus* mosquitoes. Within the Coastal Plains populations, GAN and JAG were the most genetically structured population comprising of more than 95% of cluster I, which suggest that GAN and JAG were highly differentiated from other geographic populations, possibly reflecting distinct introduction events with different geographic origins. This may be attributed to the frequent movements (in and out) of people from both the districts for trade related purposes like importation of materials etc., which in turn has modulated the genetic architecture of *Ae. albopictus,* thereby increasing the chances of transmission of arboviral diseases. Both the districts are on the verge of urbanization, which has led to the formation of ideal breeding sources, host availability, etc. that conferred an advantage to *Ae. albopictus* for rapid colonization and hence becoming more genetically structured. Similar results were obtained in La Reunion, which depicted that *Ae. albopictus* preferred to breed more in urban areas than in rural areas, thereby increasing its genetic diversity [43]. Furthermore these districts have been the epicentre for arboviral outbreaks in recent time, which can be attributed to the high genetic structuring of *Ae. albopictus* circulating in these regions. The NJ tree also supported the cluster analysis since GAN and JAG grouped together in the phylogenetic tree. In a broader view, the Coastal Plains populations were grouped together, with one exception of BAL (which grouped with ANG and DHE), while the Northern Plateau populations (MAY/KEO) and Eastern Ghats populations (KOR/MAL) grouped together in the NJ tree as another distinct clade.

*Wolbachia-*based vector control strategies to complement the existing vector control methods are currently being investigated by several researchers [44]–[47]. When a vector population consists of individuals uninfected/infected with same/different *Wolbachia* strains, their mating can be 1. compatible and produce viable offspring; 2. incompatible in both directions and produce infertile eggs (a phenomenon called bidirectional CI) or 3. incompatible in one direction while the reciprocal cross is fertile (unidirectional CI). These attributes are now being studied by many researchers with the aim of developing new technologies and strategies to achieve significant improvements in vector control [48]. Hence the data on natural infection of *Wolbachia* in mosquitoes is crucial to evaluate the possibility of utilizing *Wolbachia*-based vector control strategies. The present study based on PCR based genotyping of *Wolbachia* infection in *Ae. albopictus* mosquitoes of Orissa indicated widespread infection in the populations screened. This study revealed the infection/super-infection of two types of *Wolbachia* strains, i.e., wAlbA and wAlbB in *Ae. albopictus* mosquitoes of Orissa. Superinfection was more pronounced in Coastal Plains populations than other populations, which can thus modulate the vector dynamics for transmission of arboviral diseases in the Coastal regions. More detailed analyses revealed that 98% females were superinfected with wAlbA and wAlbB strains, which is in compliance with many studies that suggested both A and B strains in natural female *Ae. albopictus* are common and virtually fixed in a population and hence can be vertically transmitted efficiently [44], [45]. wAlbA was detected in only few *Ae. albopictus* mosquitoes, which suggests that the loss of wAlbB is hardly adaptive and hence the same vertical transmission of wAlbA+wAlbB will be maintained in the populations. The observed distribution of wAlbA and wAlbA+wAlbB infections in *Ae. albopictus* populations could be explained by the replacement of an uninfected population by the wAlbA+wAlbB superinfection [49]. Monoinfection of wAlbB was not detected in naturally occurring populations of *Ae. albopictus* mosquitoes of Odisha, which is in conformity to other studies that have documented similar findings [45], [50].

The findings of this study will provide a benchmark to the State vector control authorities on the pattern of infection of *Wolbachia* in natural *Ae. albopictus* populations of Orissa and emphasize the influence of genetic diversity of *Ae. albopictus* in different topographical regions, which will help to initiate vector control programmes. Since *Wolbachia* infection has substantial influence on host fitness, the potential impact of *Wolbachia* on host population genetics and geographical patterns is extensive. *Wolbachia* can alter host population genetics through its CI inducing behavior that causes infected females to produce more offspring than uninfected ones. Thus, our findings emphasize the need for further investigation with deeper sampling and more genetic loci in order to thoroughly elucidate the forces that shape and maintain the population structure.

## Supporting Information

Table S1 **Microsatellite primer sequences, repeat motives, size ranges and annealing temperatures used in this study.**
(DOC)

Table S2 **Analysis of Molecular Variance (AMOVA).**
(DOC)
