# Supplementary material for: Genetic Structure and Wolbachia Genotyping in Naturally Occurring Populations of Aedes albopictus across Contiguous Landscapes of Orissa, India
Source: PLoS One. 2014 Apr 8;9(4):e94094. doi: 10.1371/journal.pone.0094094 (PMC3979767; doi:10.1371/journal.pone.0094094)
Supplement: Table S1 — Microsatellite primer sequences, repeat motives, size ranges and annealing temperatures used in this study. (DOC) [file pone.0094094.s001.doc]

**Table S1. Microsatellite primer sequences, repeat motives, size ranges and annealing temperatures used in this study.**

| **Locus names** | **Accession nos.** | **Repeat motif** | **Forward sequence** | **Reverse sequence** | **Range (bp)** | **T_A_ (°C)** | **Dilution factor** |
| --- | --- | --- | --- | --- | --- | --- | --- |
| **AealbA9**  **AealbB51**  **AealbB52**  **AealbD2**  **AealbB6**  **AealbF3**  **AEDC** | **DQ366022**  **DQ366023**  **DQ366024**  **DQ366021**  **DQ366026**  **DQ366027**  **T58313** | (AC)4GCAT(AC)2TC(AC)8CCAA  (AC)2CG(AC)GT(AC)C(AC)AT(AC)  (AC)A(AC)A(AC)2 … (AC)6 …  (T)3G(T)5G(T)4GGG(AC)3  (AC)3T(AC)2AA(AC)AAA(AC)3AA(AC)AT(AC)2T(AC)2  (A)16(AC)9GC(AC)22  (AC)1AT(AC)7GC(AC)2GCAT(AC)6AG(AC)  (AC)6AT(AC)3AAAA(GC)2  (GTA)6(ACG)(GTA)3 | 5’FAM-TGGGACAAGAGCTGAAGGAT  5’VIC-TCCACGTGGTATAACTCTGA  5’FAM- GGGTCTAGAAGTAATAGCGATG  5’VIC-GAATCCCACACAGCGTCTTT  5’HEX-ATGAGGTGACCCTTTTGTGC  5’HEX-CTCGTGAGTACGTTCCGTGA  5’FAM-TGCAGGCCCAGATGCACAGCC | CTCGTTCTCTACTCTCTCCGTT  GTAGTTGTCCAATTAACATCG  GCATTCTTTGCTTCTGTTTGC  GGTCGCTTGACACCTTGAAT  AAATTTTATAGGGCCCTCGG  AGGGAAACAAGGACTTCATCA  TCCGCTGCCGTTGGCGTGAAC | 142-162  124-167  165-181  181-216  128-162  215-245  210-230 | 55.1  54.5  52.1  56.1  53.2  54  54.2 | 1/50  1/60  1/60  1/60  1/50  1/50  1/100 |
