# Supplementary material for: Genetic Structure and Wolbachia Genotyping in Naturally Occurring Populations of Aedes albopictus across Contiguous Landscapes of Orissa, India
Source: PLoS One. 2014 Apr 8;9(4):e94094. doi: 10.1371/journal.pone.0094094 (PMC3979767; doi:10.1371/journal.pone.0094094)
Supplement: Table S2 — Analysis of Molecular Variance (AMOVA). (DOC) [file pone.0094094.s002.doc]

| **Table S2. Analysis of Molecular Variance (AMOVA)** | | | | | |
| --- | --- | --- | --- | --- | --- |
|
| Source of variation | df | MSD | Variance | % variance | p |
| Between regions | 4 | 366.134 | 319.953 | 29.65 | <0.0001 |
| Among populations within regions | 11 | 214.584 | 174.815 | 14.09 | <0.0001 |
| Within populations | 185 | 886.429 | 698.583 | 56.26 | <0.001 |
